# Kuru Disease: Bridging the Gap Between Prion Biology and Human Health

**DOI:** 10.7759/cureus.51708

**Published:** 2024-01-05

**Authors:** Himanshu Kothekar, Kirti Chaudhary

**Affiliations:** 1 Anatomy, Jawaharlal Nehru Medical College, Datta Meghe Institute of Higher Education and Research, Wardha, IND

**Keywords:** kuru treatment, creutzfeldt-jakob disease (cjd), transmissible spongiform encephalopathies, human prion disease, kuru disease

## Abstract

This article explores the intriguing case of Kuru disease, a rare and fatal prion disease that once afflicted the Fore people of Papua New Guinea. Scientists are still perplexed as to the origins of Kuru because efforts to discover infectious agents like viruses have been ineffective. Initial research revealed similarities between Kuru and scrapie, a neurological disorder that affects sheep, suggesting potential similarities between the two diseases. In further research, experiments in which chimpanzee brain tissue from Kuru patients was implanted led to the development of Kuru-like symptoms in the animals, suggesting a transmissible component to the condition. Furthermore, data collected from epidemiological studies highlights a drop in Kuru transmission, especially after the Fore people stopped engaging in cannibalism, and the disease showed different incubation times that affected persons within particular age groups. Neuropathological tests in the infected brain tissue have found typical intracellular vacuoles, spongiform alterations, and amyloid plaques. According to studies, Kuru susceptibility has been linked genetically to particular PRNP gene variations. Kuru and other prion disorders have few effective treatments currently, underlining the vital need for early identification. Scientists have created sensitive detection techniques to stop the spread of prion diseases and looked into possible inhibitors. Hypochlorous acid, in particular, has shown potential in cleaning processes. Besides making great progress in understanding Kuru, there are still many unresolved issues surrounding its causes, transmission, and management. The terms "kuru disease," "human prion disease," "transmissible spongiform encephalopathies," and "Creutzfeldt-Jakob syndrome" were used to search the studies; papers unrelated to the review article were removed. Eighty-four articles are included in the review text to fully understand the complexities of this puzzling disease and its consequences for prion biology and human health; additional study is essential.

## Introduction and background

**Figure 1:**
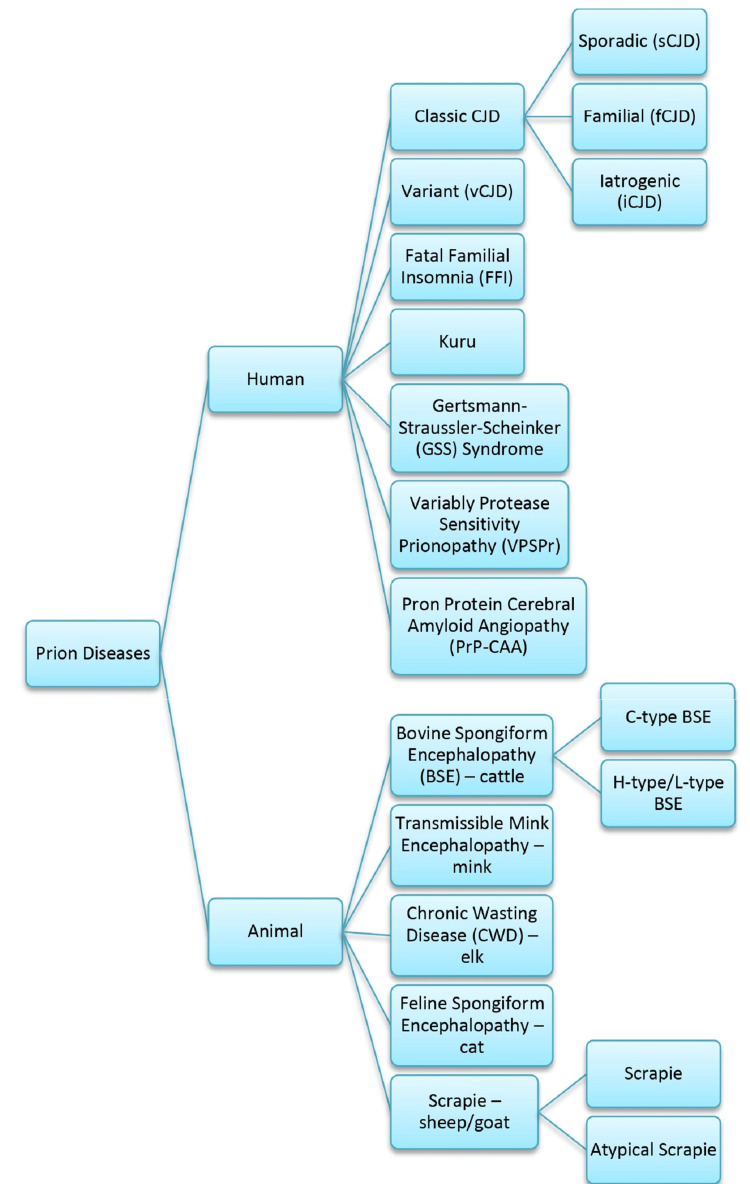
Classification of prions

Prion infections refer to a group of diseases that affect the brain that is brought on by the transformation of the normally -helical, prion-associated protein (PrPC; C represents the protein's cellular form) into an aberrant prion protein (PrPSc; Sc refers to scrapie, a type of prion disease affecting sheep and goats; PrPSc signifies the protein-rich contagious particles) [1]. Alfons Jakob recorded the first prion infection cases among individuals between 1921 and 1923 and considered that their symptoms were comparable with those of a young lady named Hans Creutzfeldt, mentioned in 1920. Jakob or Jakob-Creutzfeldt disease was the term used for the disorder for a long time [2,3]. It was once thought that "slow viruses" could have caused the previously indicated diseases, known as transmissible spongiform encephalopathies, including scrapie syndrome and Jakob-Creutzfeldt disease. Their tendency to spread and the length of time between exposure and symptom onset were factors in this hypothesis [4]. Prion disease is classified under Figure 1.

D. Carleton Gajdusek identified "Kuru" as the initial prion illness [5-14]. The illness was also called "a transmissible spongiform encephalopathy" (TSE), or slow peculiar infectious disorder, and it was the initial case of a human prion disease that had been transferred to chimpanzees. Gajdusek and Vincent Zigas reported it for the first time in two articles in 1957 [15,16]. Creutzfeldt-Jakob disease (CJD) was subsequently spread as a result of the classification of Kuru as being simultaneously infectious and neurodegenerative (not inflammatory) [17-19]. Furthermore, the idea indicated that Kuru represented a novel class of diseases brought on by a unique group of germs known as prions. The concept of conformational diseases or prionoids emerged due to research on the Kuru, which also impacted nucleation-polymerization theories [20].

"Kuru" used in the Fore language denotes shivering brought on by a common cold or fever [21-24]. The existence of Kuru encloses most Fore village people claim that the problem has existed for a long time, but they quickly point out that things have gotten worse recently and that Kuru didn't even exist when the initial sources were children [18]. According to the Fore records, Kuru originally arose around 1900 in the nearby Keiagana hamlet of Uwami before moving on to the North Fore around 1920. By 1930, it had made its way along the southern boundary and was within the center of South Fore at Wanitabe. When it originally started in Wanikanto in 1927, it shifted northwest towards Paigata and Miarasa. The disease emerged in certain southern and southeastern regions as late as the 1940s, with the earliest instances being in the 1930s in Purosa, six miles south of Wanitabe [8]. When the symptoms of Kuru initially occurred, people fed the patients with the bark part of the casuarinas tree as a homeopathic remedy that provided very minimal comfort because they believed the symptoms resembled the quills of the cassowary and the waving fronds of the casuarinas tree. Due to the affected women's excessive laughter, the illness was also frequently referred to as "Negi nagi," a Fore phrase that means a silly or dumb person [17]. Incidence of Kuru grew throughout the 1940s and 1950s, approaching the 35/1000 death rate in several villages with 12,000 or more Fore people living there [15,17,25-28]. The South Fore had a female-to-male ratio of 1:1.67 as opposed to the unaffected Kamano population's 1:1 ratio, which was warped by the death rate. In South Fore, this ratio rose to 1:2 or even 1:3. Gajdusek even estimated that there were 1676 fewer women in the population than there were males [29].

In 1955, Zigas learned about Kuru, and two years later, in DC. Gajdusek joined him. In the 2000s, He questioned Gajdusek about the origin of the theory that cannibalism may be used to disseminate Kuru. He stated in reply, "Even a drunken man would think that a disease common to cannibals could only have transmitted through consuming corpses". Gajdusek made this declaration around half a century following the discovery of Kuru. Lindenbaum and Glasse were the first to explicitly publish the theory that Kuru spreads through cannibalism [8]. Although postmortem investigations did not show any perivascular cuffings or other signs of inflammation-related neurological illnesses based on epidemiological research, it is nevertheless thought that the source of Kuru was likely pathogenic.

Additionally, the patients had no cerebral spinal fluids, pleocytosis, excessive protein levels, or signs or symptoms of meningoencephalitis (coma, convulsions, or fever). No hints were offered by the genetic investigations conducted at the time or by studies of the environment [21,30-38]. In order to address potential resurgence and comprehend the long-term effects of the disease, ongoing research is needed. This includes monitoring and evaluating existing kuru cases, conducting long-term follow-up studies on affected individuals, investigating genetic and environmental risk factors, developing preventive measures, collaborating internationally, raising public awareness, and developing preventive measures [39].

## Review

Search methodology

**Figure 2:**
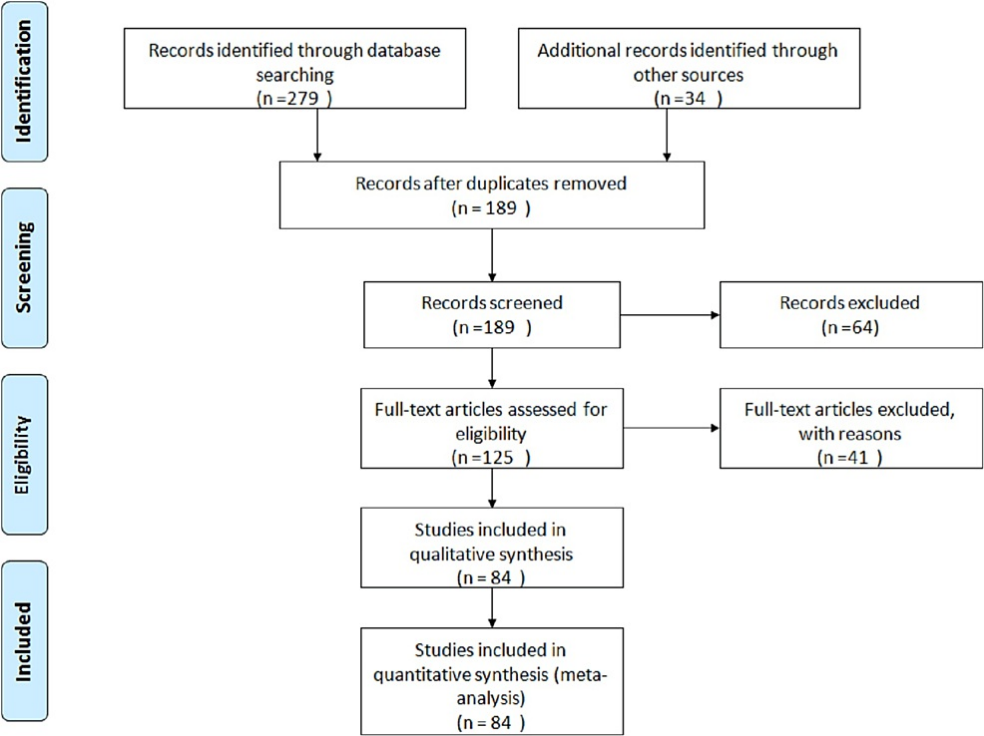
The PRISMA flow diagram represents the inclusion and exclusion criteria used

The literature of the following review is gathered from the two resources, namely, PubMed and Google Scholar. The studies were searched, which included the keywords "kuru disease," "human prion disease," "transmissible spongiform encephalopathies," and "Creutzfeldt-Jakob syndrome," and those articles that are not related to the review article were excluded. The length (years) of the article "Kuru Disease: Bridging the Gap between Prion Biology and Human Health - A Review" is drawn from very old articles to offer a historical perspective and chart knowledge development about Kuru disease. This makes it possible to thoroughly analyze the disease's effects on human health over time and gain insights into its course and long-term effects. The study's source materials, from 1920 to 2019, offer a thorough historical review of Kuru, its clinical presentations, epidemiology, genetic factors, and experimental studies. Of particular note is how knowledge has evolved over almost a century. The selection of articles utilized in the following review article is depicted below in Figure 2.

Etiology

The efforts to detect microorganisms, including viruses, by utilizing tissue cultures or embryonated hen eggs to transfer Kuru to laboratory rodents were unsuccessful. No viable explanation could be produced despite extensive genetic analyses, research into possible nutritional inadequacies, and assessments of exposure to environmental toxins [30,32]. A letter from William Hadlow, a US veterinarian pathologist working at Hamilton, Montana's Rocky Mountain Laboratory, was sent to Gajdusek on July 21, 1959, when he was still in New Guinea. In the note, Hadlow emphasized the resemblances between Kuru and scrapie, a slowly infecting neurological disorder of goats and sheep that has been widespread in the UK since the eighteenth century, and it was first clinically transferred in 1936. He encloses a replica of a note that he wrote the Lancet's editor pointing away the resemblance between scrapie and kuru plaques after seeing photos exhibiting kuru plaques in the London display at the Wellcome Medical Museum [40-42].

Innes, a veterinary neuropathologist, had a comparable finding when visiting the Gajdusek lab (Gajdusek-telephone conversation, 2008). Hadlow identified intracellular vacuoles as the neuropathological alterations that initially caught his eye some 40 years ago in remembrance of that crucial finding. In 1898, Besnoit and his coworkers published the first description of such intracellular vacuoles in scrapie [27,43,44]. None of the monkeys given the inoculation showed symptoms suggestive of a persistent neurological disorder before the recent emergence of the illness in two chimpanzees, even though some suffered severe illnesses throughout the observation period. Gibbs and Gajdusek provided this information in an update to their 1965 monograph, "Slow, Latent, and Temperate Virus Infections" [8]. The initial of the two received an inoculation twenty months ago via a suspension of preserved cerebral tissue taken from a kuru patient who has since developed gradually accompanying immobilized cerebral symptoms of tremor and ataxia; the other one received an inoculation identically using an amalgamation of tissue from the brain of an additional kuru patient who developed mild wasting lassitude and some tremor that developed as progressive symptoms Twenty-one months after that. It is unknown whether these disorders result from inoculation or are spontaneous [45].

Epidemiology

**Figure 3:**
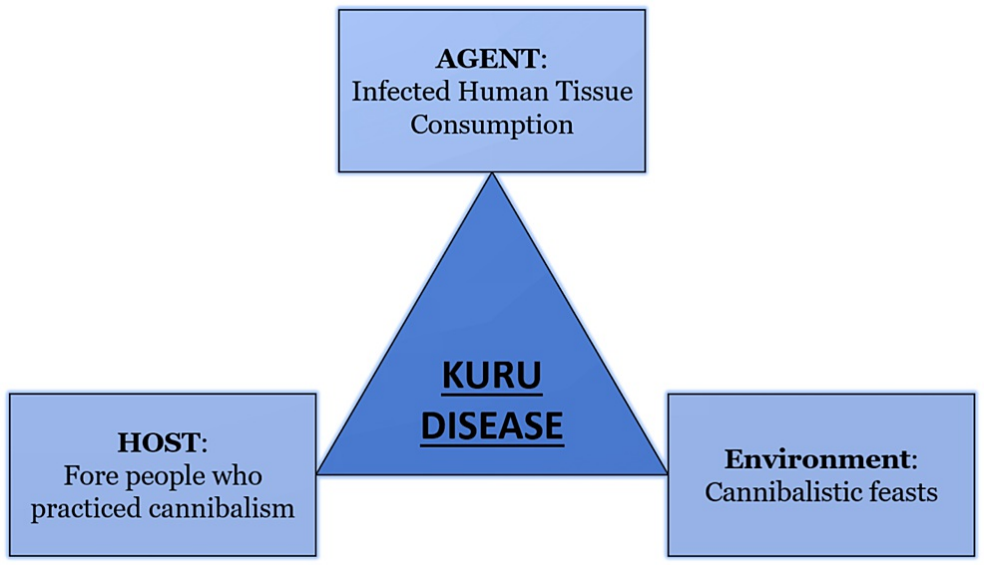
Epidemiological triangle for the Kuru disease

Kuru transmission among children came to a stop in the 1950s when cannibalism among the Fore people was abolished. This is supported by the fact that there aren't many Kuru cases among people in the South Fore region who were born after 1954 and that the average age of patients is gradually rising. Furthermore, it was noted that brothers with Kuru frequently passed away at a similar age, suggesting that they probably developed the condition at a similar age, although not necessarily at the same time. In males, the disease took an average of 3 to 6 years to progress, with maximum incubation periods of up to 10 to 14 years. If affected brothers acquired Kuru at the same age, the earliest age of exposure for males could range from 1 to 6 years [46-48]. Klitzman et al., in the 1940s and 1950s, analyzed groups of patients who engaged in kuru cannibalism and proved that Kuru was spread through cannibalism. These clusters appeared in three. Ob and Kasis, two brothers from the North Fore hamlet of Awande, got Kuru in one of the three cases in 1975, twenty-one or twenty-seven years, depending on whether the contact was sooner or afterward. They participated in two cannibalistic feasts [8]. The epidemiological triangle for the Kuru disease is represented in Figure 3.

Transmission

After intracerebral inoculation of brain suspension from kuru patients, a kuru-like condition in chimpanzees occurred that closely resembles kuru in humans. Seven chimpanzees have developed the condition, and three more chimpanzees have acquired it through the transmission of affected individuals' brain tissue [49-52]. The seven chimpanzees first displayed symptoms of the condition about 18 to 30 months after receiving an intracerebral injection of 10% brain suspension from six distinct Kuru patients. Five of these chimpanzees received brain tissue from various Kuru patients, whereas the other two received brain tissue from the same Kuru patient [17]. Gajdusek's theory is supported by several variables. First off, neither clinically nor pathologically, the illness he experimentally created does not reflect any known disorder in chimpanzees, other apes, or monkeys. Second, neither the unvaccinated nor the chimpanzees who received a different type of vaccination from our primate colony had any neurological diseases. Thirdly, his theory is supported by data from human brain tissue linked to various neurological illnesses. Furthermore, regarding the available scientific evidence and the pathological traits, the syndrome he caused in chimpanzees is very similar to kuru in humans. Last but not least, after an equally protracted incubation period, seven of eight chimpanzees that received an injection of kuru cerebral tissue showed symptoms of the disease, supporting his theory [53].

Cannibalism

Endocannibalism- the consuming of family members- was an element of bereavement rites in the South Fore, in comparison with exocannibalism, which was practiced in the North and included consuming rivals. This had ramifications for the transmission of the disease. Cannibalism stopped in the North Fore by the 1950s, but it was still carried out covertly in the South Fore, who claimed to have kept on concealing and consuming their dead relatives throughout the 1950s [54,55]. All parts of a person were regarded as suitable for consumption by humans, except for the gall bladder, which was deemed to be extremely bitter. The deceased's maternal kin dissected the body in the former sugarcane garden. They cut apart both the extremities of the body to extract the muscular tissue after first removing the hands and feet. The gall bladder was prevented by opening the chest and belly. After breaking the skull to take out the brain, they decapitated the head. The brain, viscera, and meat were all consumed. Cracked bones were used to extract the marrow, and the ground-up remains of the cartilage were occasionally prepared in greenish leafy plants [8]. Occasionally, sorcerer identification rites were carried out. According to one technique, the presence of the suspect close to a dead body when fluids began to escape from it proved their guilt, even if the person had already passed away. Another method involved putting the name of a possible sorcerer in the bamboo cylinder that contained a recently killed possum and hair from a kuru victim. It was thought that the possum meat would reveal the sorcerer's location if it was still intact. These divination practices typically mentioned certain homes rather than naming specific people [8].

Neuropathology

In 1959, Klatzo et al. published the first examination of kuru neuropathology (12 cases) [56]. The dispersion of Nissl material and intracytoplasmic vacuoles are comparable to the ones previously observed in scrapie. Neurons were undersized, hyperchromatic, or pale, and some of the neurons within the striatum, along with the cerebellum's Purkinje cells, developed a "moth-eaten" appearance due to vacuolation. A sponge-like alteration was noticed [8]. The most significantly afflicted areas of the cerebellum were the paleocerebellar structure (vermis and flocculo-nodular lobe), whereas the corticospinal and spinocerebellar pathways have the most significant spinal cord damage.

Furthermore, the expansion of astrocytes and microglia was widely used; the last one was developed star and manifested as macrophages (gitter cells), rod- or amoeboid-like cells, or microglia. In 10 out of 12 cases, myelin deterioration was seen. Though "small spongy spaces" were seen in 7 out of the 13 patients examined by Beck and Daniel, Klatzo et al. did not recognize the importance of vacuolar alterations [56-58].

The existence of many plaques of amyloid, sometimes known as "kuru plaques”, was considered to be the most notable neuropathologic characteristic of kuru and was discovered in six of the twelve cases analyzed by Klatzo et al. and in "about three-quarters" of the thirteen instances examined by Beck and Daniel [56-65]. The emergence of variant CJD, a unique form of the disease marked by many plaques of amyloid, including "florid" or "daisy" plaques-kuru plaques encircled with spongiform vacuolated ring-raised interest in kuru pathology once again. A few publications that reevaluated old information were published to achieve this. They looked at the situation of an early man with a kuru victim known as Kupenota, belonging to the South Fore geography using PrP-immunohistochemistry, the cerebral structure caused the infection to spread to chimpanzees. McLean, with his team members, looked at 11 kuru cases. The deep layers (III-V) of the cingulate, occipital, entorhinal, and insular cortices, as well as the subiculum, all contain the characteristic spongiform alteration discussed in the two recent publications [65-68].

Signs and Symptoms

**Figure 4:**
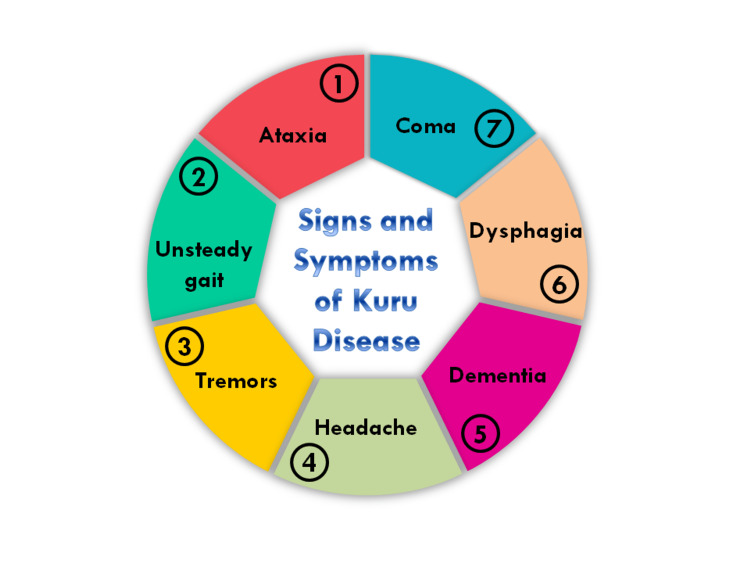
Signs and symptoms of Kuru disease

The signs and symptoms that are used to predict Kuru Disease are explained in the following Figure 4.

Molecular biology and genetics of Kuru disease

It was initially found that two cases of Kuru included 129^Met Met^. Furthermore, research revealed that people with the genotypes 129^Met Met^ and 129^Val Val^ were both susceptible to Kuru; however, people with the 129^Met Met^ genotype were more prevalent in the younger age category, while those with the 129^Val Val^, 129^Met Val^ genotypes were more prevalent in the far older age category [69-74]. On the other hand, those that survived the outbreak had a nearly complete lack of 129^Met Met^ homozygotes. All subjects in contemporary cases studies conducted by Lantos et al. and McLean et al. were 129^Met Met^ homozygotes. A strong correlation among Kuru as well as two SNPs in codon 129, along with two additional SNPs beneath the RARB (retinoic acid receptor beta) and STMN2 (SCG10 gene) genes, according to recent genome-wide analyses [67,68,71,75].

The Collinge group carried out the genetic variant identification of kuru patients. The classification is based on how easily the PrPSc de-, mono-, and diglycosylated clusters move along the electrophoresis after being digested by proteinase K. They discovered the four main kinds of PrPSc. Human PrPSc types 1 and 4 exclusively appear in populations whose PRNP gene has the codon 129^Met Met^; type 3 is visible in populations whose PRNP gene has at least one Val at this codon; and type 2 is seen in all codon 129 variations [76-78]. The agreement between proponents of either categorization has not yet been reached. Another classification solely considers two PrPSc kinds; the kuru samples had either type 2 (129^Met Met^) or 3 (129^Val Val^) PrPSc trends, and their glycoform ratios were comparable to those of sporadic CJD rather than usual for vCJD. The abnormality of "b" type, or grittier chambers located in more deeply layered structures of the subcortical levels of the cortex in the brain, was seen in monkeys that had been vaccinated with Kuru, sCJD VV2, and sCJD MV 2K [79-82]. At first, it was thought healthy wild mice weren't susceptible to the kuru virus. However, later studies showed it can spread to CD-1 mice, resulting in unique clinical and neuropathological traits in infected animals. Notably, the fascinating possibility of the kuru agent moving into the bloodstream is increased by the high concentration of PrPSc in the dendritic follicle cells of the spleen. PrPSc has been found in dendritic follicle cells in other TSE. Collectively, these findings imply that Kuru is distinct from both sporadic and variant CJD [83].

Treatment and diagnosis

**Figure 5:**
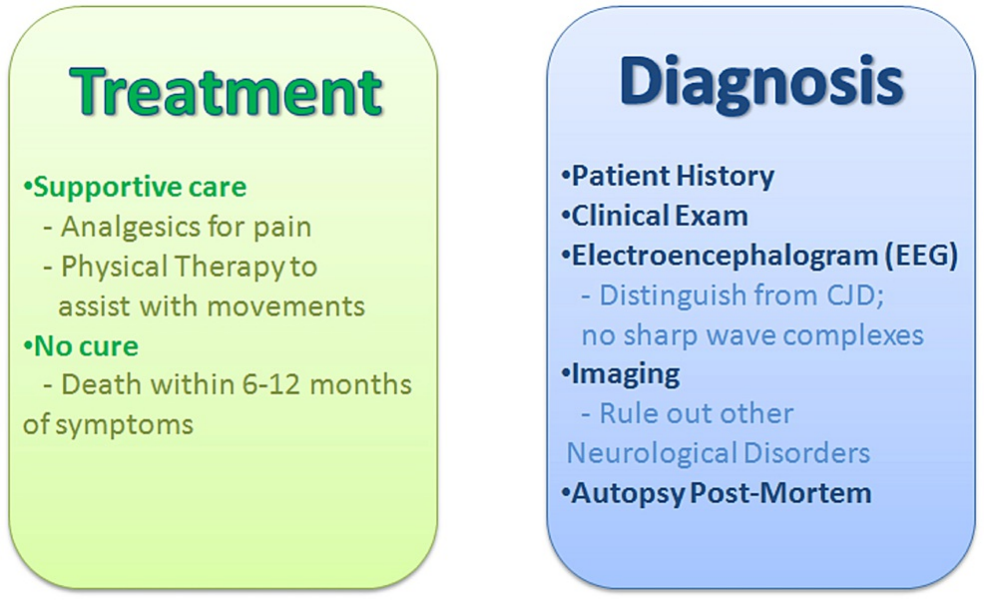
Treatment and diagnosis of Kuru disease CJD: Creutzfeldt-Jakob disease

One research studies mentions that some RML scientists developed the Real Time-Quaking Induced Conversion (RT-QuIC) assay, a rapid and hyper-sensitive detection assessment. It is used at many facilities across nations to identify CJD and enhances formerly indistinguishable levels of prions to the point at which their presence is easily distinguished utilizing cell-free prion protein transformation reactions. Researchers at the NIAID have examined thousands of substances utilizing either cell-free or cell-based assays and have discovered 100s of chemicals that prevent the development of the peculiar type of prion protein. If therapies are started shortly after infection, additional testing of the strongest inhibitors has discovered some that can extend rodents' lifespan. Hypochlorous acid, a chemical well known for its capacity to fight viral and bacterial diseases, has also been found to successfully eradicate prions, according to research at the NIAID's RML. Hypochlorous acid is a potentially useful disinfection treatment for hospitals in the fight against prions because it offers no risk to human health [84]. The treatment and diagnosis of Kuru disease are depicted below in Figure 5.

## Conclusions

The review looks into Kuru's complex etiology, underlining the difficulties in determining its etiological agents and making fascinating analogies with scrapie, a related neurological condition. Researchers now have a better grasp of the biology of the disease because of neuropathological investigations from past studies that identify unique abnormalities such as intracellular vacuoles, spongiform alterations, and amyloid plaques in the brain tissue of affected people. As a result of the Fore people's cannibalism being forbidden, epidemiological studies highlight a decrease in Kuru transmission. Additionally, the disease's epidemiology is complicated by its varied incubation periods and age-specific symptoms. Further genetic study reveals associations between particular PRNP gene variations and vulnerability to Kuru, illuminating the underlying genetic causes. Despite advancements, Kuru and other prion disorders are still without effective treatments, underlining the significance of early detection. Research is ongoing for its overall effect on human biology and health to discover proper methods of diagnosis and treatment.
